# Comparative evaluation between hypericin (hypiran) and fluoxetine in treatment of companion dogs with tail chasing

**Published:** 2015-06-15

**Authors:** Bahman Mosallanejad, Hossein Najafzadeh Varzi, Reza Avizeh, Mahdi Pourmahdi, Fatemeh Khalili

**Affiliations:** 1 *Department of Clinical Sciences, Faculty of Veterinary Medicine, Shahid Chamran University, Ahvaz, Iran; *; 2 *Department of Basic sciences, Faculty of Veterinary Medicine, Shahid Chamran University, Ahvaz, Iran; *; 3 *Department of Food Hygiene, Faculty of Veterinary Medicine, Shahid Chamran University, Ahvaz, Iran; *; 4 *Graduated from Faculty of Veterinary Medicine, Shahid Chamran University, Ahvaz, Iran.*

**Keywords:** Ahvaz, Dog, Fluoxetine, Hypericin, Tail chasing

## Abstract

The aim of the present study was to compare the effects of hypericin and fluoxetine in the treatment of companion dogs with tail chasing in Ahvaz district. In the present survey, eighteen dogs with tail chasing were assigned into three equal groups for a three-year period. The dogs were randomly classified based on different treatment groups. During 15 weeks, dogs of group A were given 0.05 mg kg^-1 ^hypericin orally and dogs of group B received 1 mg kg^-1 ^fluoxetine, orally. The group C was the control group. Changes in signs of tail chasing were weekly reported by the owners or a veterinarian. Treatment periods were assessed in five intervals: weeks 1-3, 4-6, 7-9, 10-12 and weeks 13-15, respectively. Hypericin (group A) was significantly more effective in the treatment of tail chasing compared with fluoxetine (group B), (*p* = 0.043). Statistical analysis revealed a significant difference in each group between weeks 1-3 (X^2 ^= 8.8, *p* = 0.01), 4-6 (X^2 ^= 9.1, *p* = 0.01), 7-9 (X^2 ^= 7.4, *p* = 0.03), 10-12 (X^2 ^= 10.4, *p* = 0.005) and 13-15 (X^2 ^= 12.5, *p* = 0.002). Improvement of behavior in the dogs of group A was significant compared with group B, between weeks 10-12 (X^2 ^= 5.4, *p* = 0.02) and 13-15 (X^2 ^= 7.2, *p* = 0.007). In conclusion, our survey showed that hypericin was more effective than fluoxetine in controlling signs of tail chasing.

## Introduction

Behavior problems are common in among domestic dogs. It has been estimated that up to 90% of dogs may exhibit behaviors that their owners find unacceptable.^1^ Canine compulsive disorder is a syndrome of abnormal behaviors that affects many breeds. The most susceptible seem to be the hunting breeds. Genetic predisposition is a major factor in whether a dog might develop canine obsessive-compulsive disorder.^2^ Examples are persistent tail-chasing, acral lick granuloma and self-mutilation.^3^ Compulsive tail chasing in dogs is a stereotypic or obsessive-compulsive disorder. It may be seen following physical trauma, surgery, or medical illness, as well as it may represent an epileptic episode or a biochemical disturbance at the level of neurotransmitter systems. Scientific literature exclusively refers to tail-chasing, when the behavior is not necessarily focused towards the tail, because it can indicate disorders with varying severity.^4^^-^^6^ Recently, similarities in the clinical signs, development, and response to pharmacological treatment of compulsive behavior patterns in companion animals and humans have been recognized.^7^^,^^8^

 *Hypericumperforatum*is used inmany countries including Iran for the treatment of depression, and its efficacy has been confirmed in several clinical studies.^9^ The therapeutic action of *Hypericum*is comparable to that of tricyclic antidepressants. Several behavioral studies support the antidepressant activity of *Hypericum *extracts,^10^^-^^11^ which has been attributed to the phloroglucinol derivative hyperforin, and to several flavonoids.^12^ In the present study, we examined the effects of administration of hypericin (Hypiran; an active constituent of St. John’s wort) and fluoxetine in the treatment of companion dogs with tail chasing in Ahvaz district, southwest of Iran. Fluoxetine, a selective serotonin re-uptake inhibitor widely used in human medicine and has proven efficacy in treating anxiety disorders in people. It is also effective in treating aggressions, obsessive-compulsive disorders, generalized anxiety and separation anxiety in companion animals.^13^^,^^14^ Flouxetine has been successfully used in human and canine obsessive compulsive disorders.^8^ Hypericin appears to be safe when used in dogs, and side effects have not been reported since. Tail amputation has no reported success, and the problem can sometimes be so intractable and distressing for the owners, that dogs are euthanized.^10^

Epidemiological studies are important to understand the risk factors for behavior problems and thus the best preventive measures. However, results can vary depending on the geographical area and the source of data. Many owners who are reluctant to take care of their dogs might be willing to keep them if there is an improvement in their behavior. Prevention of undesirable behavior is also important from a public health standpoint, particularly in relation to aggression.^15^^,^^16^ There are few reports of research on treatment for tail chasing in dogs. Therefore, the objective of the present study was to compare the effectiveness of hypericin and fluoxetine in the treatment of dogs with tail chasing.

## Materials and Methods

**Animals. **Eighteen dogs with tail chasing were selected in the present study between 2010 and 2012. In this research, census data were used. Tail chasing was diagnosed on the basis of the dog’s behavioral history (age, frequency and duration of bouts since onset, intensity of the behavior, history, and current or previous medical conditions), clinical signs and results of laboratory features. For this reason, the eighteen companion dogs referred to the veterinary hospital of Shahid Chamran University of Ahvaz met the inclusion criteria. The dogs were classified based on different treatment groups (Table 1). Affected dogs had to have tail chasing bouts for a minimum of 30 sec per bout to be included in this survey.^8^ The owner responses were scored as ordinal scales. They were allocated into three equal groups (A, B and C). During 15 weeks, dogs of group A were given 0.05 mg kg^-1 ^hypericin (Pursina Pharmacy, Tehran, Iran) once daily and dogs of group B received 1 mg kg^-1 ^fluoxetine (Pursina Pharmacy, Tehran, Iran) once daily orally. In control group (C), an equal volume of normal saline 0.9% was administered. Changes in signs of tail chasing were weekly reported by the owners or the presence of a veterinarian in dwelling. Treatment was assessed in five intervals: weeks 1-3, 4-6, 7-9, 10-12, and 13-15, respectively. Doberman pinscher, Terrier and German Shepherd dogs were participated in this survey.

**Table 1:** Breeds, sex and age of the studied dogs in three groups A (0.05 mg kg^-1 ^hypericin), B (1 mg kg^-1 ^fluoxetine) and C (normal saline).

**Characteristics**	**Hypericin group (n = 6)**	**Fluoxetine group (n = 6)**	**Control group (n = 6)**
**Breeds**	DP (n = 2) GS (n = 3) T (n = 1)	DP (n = 1) GS (n = 3) T (n = 2)	DP (n = 3) GS (n = 2) T (n = 1)
**Sex**	Male (n = 4) Female (n = 2)	Male (n = 3) Female (n = 3)	Male (n = 4) Female (n = 2)
**Mean of age (months)**	12 (10-24)	16 (12-28)	14 (8-22)

DP = Doberman pinscher, GS = German Shepherd, T = Terrier.

**Study design. **The dogs were 8 to 28 months and vaccinated using Biocan DHPPiL+R (Bioveta, Ivanovice, Czech Republic). They were fed a home-made diet mostly. This study was performed under the control of the Iranian Society for the Prevention of Cruelty to Animals. The owners were advised to exercise their dogs 20 min per day.

**Sampling. **Blood samples were collected from cephalic or saphenous vein with and without EDTA for complete blood count (CBC) and serum concentrations of urea, creatinine, total protein, albumin, globulin, and glucose, and activities of ALP, ALT and AST. Normal values were referred to Tilley and Smith.^17^ Results of the clinicopathologic testing and physical examination were used to exclude dogs with seizure disorders, diabetes mellitus, liver, eye and cardiac disease, neuritis, ectoparasite infestations, anal sac diseases and pruritus.

**Scoring of treatment. **A description of tail chasing (such as number and intensity of episodes) was brought before challenge, to make it clear to the owner. The most important questions asked, for this behavioral problem, are shown in Table 2. The response to treatment was evaluated and monitored weekly by telephone conversation or personal contacts (by only one person). Score sheets included whether the owner had observed any adverse effects and, if so, what kind of effects had been seen, how many hours the owner had spent with the dog that day, the number of episodes of compulsive behavior the owner had witnessed that day, the duration of the longest observed episode, and whether the behavior ended on - or because it was interrupted.^10^ Similar to the research of Yalcin, the owners were asked to assign a score on the basis of the following ordinal scale for improvement in behavior: 0 = no change was observed, 1= minimal improvement, 2 = moderate improvement, 3 = marked improvement and 4 = substantial improvement.^8^ Response to treatment was evaluated for the following five intervals: weeks 1-3, 4-6, 7-9, 10-12 and 13-15.

**Statistical analysis. **Dogs were grouped based on different treatment groups and scoring of treatment to determine whether these factors were associated with the status of disease. Data were analyzed by Friedman test followed by Wilcoxon signed rank test in each group. The Kruskal Wallis test followed by Mann Whitney U Test was used to compare score differences between the three groups using SPSS (Version 17; SPSS Inc., Chicago, USA). For comparison of scores between each treatment section in each group, results were analyzed using the Friedman test. Improvement in behavior for sequential weeks computed as score differences was analyzed by using the Kruskal Wallis test. Differences were considered significant when *p* < 0.05.

**Table 2:** Behavior questionnaire for tail chasing in the studied dogs (the most important questions).

On average, how much time on a day does the dog spend chasing its tail? On average, how long does one tail chasing bout last? How does the tail chasing episode end? In what state is the dog? You can specify more than one option. Have you ever tried to stop the behavior? Do you give medication for your dog because of this behavior? If yes, please specify the medication: While behaving, does the dog react to its name, or other commands? Is there any physical injury to the dog’s tail due to tail chasing? Is the dog spayed/neutered? At what age was your dog separated from its mother? When your dog was born, was there any complications etc. with the birth? Do you give your dog(s) extra vitamins/dietary supplements? How many hours/minutes does your dog get exercise in a typical day? How much does your dog spend alone in the house/kennel during the average working day? Can you think of any other important information that might be related to tail chasing behavior? Does your dog have any of the following diseases such as diabetes, epilepsy, allergies, demodicosis, hypothyroidism, pancreatic insufficiency, liver malfunction, or something else?

## Results

Responses to treatment in the three groups (A, B and C) are shown in Table 3. We compared these values among groups to determine the difference of improvement in behavior. Repeated score measures of treatment sections were significantly different for all groups (A: X^2^ = 23.40, *p* < 0.001, B: X^2^ = 16.60, *p *= 0.002, and C: X^2^ = 15.5, *p* = 0.004). In comparison between two drugs, hypericin (group A) was significantly more effective in the treatment of tail chasing than fluoxetine (group B, *p* = 0.043). Statistical analysis revealed a significant difference in each group between weeks 1-3 (X^2^ = 8.80, *p* = 0.01), 4-6 (X^2^ = 9.10, *p* = 0.01), 7-9 (X^2^ = 7.40, *p* = 0.03), 10-12 (X^2^ = 10.4, *p* = 0.005) and 13-15 (X^2^ = 12.50, *p* = 0.002). In different times, unlike of group B, group A had a significant difference with group C. Improvement of behavior in the dogs of group A was significant compared with group B, between weeks 10-12 (X^2^ = 5.40, *p *= 0.02) and 13-15 (X^2^ = 7.20, *p* = 0.007). Results of CBC and biochemical profiles were in normal range in all dogs, except for cholesterol and triglyceride that were above in most of them (14 out of 18; 77.78%).

Male dogs (n = 11) were more often affected than females (n = 7). Concerning the breed, German Shepherd dogs (n = 8) and Doberman pinscher (n = 6) were overrepresented. Only in three out of 18 dogs, tails were bandaged because of injury. Six dogs chased their tails in the presence of their owners and only stopped this behavior when their attention was focused on another object. The other dogs did not stop. Drugs affecting gastrointestinal motility including anti-emetics, anti-diarrhea medication or drugs affecting urinary function including diuretics were not administered during the trial. Worsening of behavior or adverse effects was not noted in any case.

**Table 3:** Response to treatment groups A (0.05 mg kg^-1 ^hypericin), B (1 mg kg^-1 ^fluoxetine) and C (normal saline) according to weeks.

**Interval**	**Groups (n = 6)**	**Treatment scores** *				
		**0**	**1**	**2**	**3**	**4**
**Weeks 1-3**	A	2	4	-	-	-
	B	1	5	-	-	-
	C	6	-	-	-	-
**Weeks 4-6**	A	-	2	4	-	-
	B	1	4	1	-	-
	C	4	2	-	-	-
**Weeks 7-9**	A	-	1	3	2	-
	B	1	2	2	1	-
	C	2	4	-	-	-
**Weeks 10-12**	A	-	-	1	1	4
	B	1	1	2	2	-
	C	1	4	1	-	-
**Weeks 13-15**	A	-	-	-	-	6
	B	1	-	2	2	1
	C	1	4	1	-	-

* Treatment scores: 0 = No change, 1 = Minimal improvement, 2 = Moderate improvement, 3 = Marked improvement, 4= Substantial improvement. Improvement of behavior was significant, between weeks 10-12 (X^2 ^= 5.4, *p* = 0.02) and 13–15 (X^2 ^= 7.2, *p* = 0.007) between groups A and B.

## Discussion

Despite the perceived known nature of the behavior and its potential severity in clinical cases, the in-depth knowledge is limited about tail chasing in dogs. The usual treatment for compulsive tail-chasing is drug therapy combined with behavioral therapy, such as increased owner attention and walks; the drugs may treat the clinical signs but behavioral change addresses the cause of the problem.^8^ In the present study, comparison of treatment scores showed that there were significant differences between group A and controls, as well as statistical difference was found between the two treatments (A and B). We concluded that while the two drugs were successful in the treatment of tail chasing, hypericin was significantly more effective and well-tolerated in controlling signs of obsessive-compulsive disorders, and we propose hypericin for treatment of tail chasing in dogs. Findings of this study may aid in recognition and treatment of compulsive tail chasing. Tail chasing may be associated with elevations of serum cholesterol in dogs.^8^ In our survey, high serum cholesterol and triglyceride levels may be used as biochemical parameters of compulsive tail chasing in clinical settings. Further studies are needed for a thorough analysis of the correlation between cholesterol and triglyceride levels in compulsive disorders in dogs. Anxiety, obsessive-compulsive disorder, and depression are common problems for pet animals. Because the development of drugs that alleviate these conditions is ongoing, there are many medications on the market for these behavioral problems. Increasing serotonin in the brain means less anxiety and a happier attitude. By inhibiting the brain’s system for removing serotonin, selective serotonin reuptake inhibitors cause serotonin to linger potentiating its effects.^7^^,^^13^

Onset of tail chasing between 3 and 12 months of age in eight sexually intact dogs suggested that hormonal changes associated with puberty could have been involved in triggering this condition similar as in human beings.^18^ Although tail chasing can be observed in several breeds of dogs, there are reports in the literature that the highest proportion of preservative tail chasing was observed in toy breeds (56.00%), followed by crossbreeds (43.00%) and terriers and working dogs (42.00% of both).^7^^,^^8^^,^^13^ The most affected breeds in our study were German Shepherd, and Doberman pinscher dogs.

The association of identifiable environmental, physiologic, or psychological experiences with onset of tail chasing in dogs have suggested that anxiety resulting from stress, conflict, boredom or environmental changes may have been a contributing factor.^13^ A limited number of studies are available concerning with fluoxetine for treatment of tail-chasing in dogs. In a report by Yalcin, clomipramine and fluoxetine seem to be equally effective in the treatment of tail chasing. Treated dogs responded well to the drugs and both drugs did not show superiority over each other. German Shepherd dogs and Anatolian sheepdogs were overrepresented. In all four intervals improvement of tail chasing did not differ significantly between clomipramine and fluoxetine. Improvement of behavior in the clomipramine group was significantly better than in the placebo group between weeks 1-3 and 4-6 and between weeks 7-9 and 10-12. Furthermore, there was a significantly better improvement in the fluoxetine group between weeks 7-9 and weeks 10-12 when compared to the placebo group.^8^ In the present study, improvement of behavior in the group A dogs was significant compared with group B, between weeks 4-6 and 7-9 and between weeks 10-12 and 13-15.

Khoshnegah *et al.* evaluated prevalence and risk factors of behavior problems in companion dogs in Ferdowsi University of Mashhad, Iran. The majority of respondents (85.60%) stated that their dog exhibited at least one behavior problem. The main behavior problems reported by owners were excessive activity (34.00%), inappropriate elimination (33.06%), fearfulness (30.20%), aggression towards unfamiliar people (26.10%) and destructiveness (25.50%).^19^ In another survey by Mashhadi Rafiei in Tehran, 47.10% had behavioral problems. Among 74 dogs, 25.00% had separation anxiety, 20.00% aggression, 7.90% other problems like excessive barking, digging, jumping on people and so on, 5.70% obsessive-compulsive disorder and 5.00% phobia.^20^ In research by Moon-Fanelli, of the 18 dogs, 15 were treated with clomipramine within the recommended dosage range, and three dogs required treatment at a slightly higher dosage range to control tail chasing. After 1 to 12 weeks of treatment, 9 of 12 (75.00%) dogs were reported to have a 75.00% or greater improvement (reduction) in tail chasing. It was recommended that dogs should be given clomipramine orally at a dosage of 1 to 2 mg kg^-1 ^of body weight every 12 hr.^7^ In a retrospective study of 103 dogs with various manifestations of compulsive disorder, clomoipramine was found to be significantly more effective than amitriptyline.^21^ They used fluoxetine in a dosage of 1 to 2 mg kg^-1 ^daily for an 8-week open trial on five dogs with acral lick dermatitis (ALD), and three dogs showed substantial improvement.^22^ In another study using fluoxetine in cases of obsessive compulsive disorder manifested as canine ALD a 50.00% success rate was reported.^23^ In a double-blind, randomized, placebo-controlled trial on the use of fluoxetine it was demonstrated fluoxetine was effective in the treatment of ALD in dogs.^24^ Fluoxetine was compared -with fenfluramine in 14 dogs with ALD and found that improvement on fluoxetine was significantly greater than fenfluramine.^4^ It was reported lethargy and decreased appetite as adverse effects during fluoxetine treatment in 31 dogs with compulsive disorders; nevertheless their results suggested that fluoxetine may be efficacious in the treatment of compulsive disorders in dogs.^25^ As a result of the present study, it was concluded that hypericin could be used more effectively than fluoxetine in the treatment of dogs displaying tail chasing.

Undesirable medical events (gastrointestinal and neurologic signs) have been seen in dogs with separation anxiety treated with clomipramine.^26^ We did not see any evidence of adverse effects or worsening of behavior in dogs treated with hypericin and fluoxetine, of course this may be related to insufficient observation by the owners. In conjunction with appropriate management changes, hypericin administration appears to be an effective treatment for tail chasing. The best prevention is to give the animal adequate attention and exercise, a suitable environment and carefully monitoring.^7^ Future research can record more details about the clinical signs: for example, details of tail-mouthing behavior can indicate tail or hindquarter discomfort, and persistently chasing in one direction can help diagnose compulsivity. It will also be necessary to determine what really triggers tail-chasing, to obtain meaningful prevalence of pathological and non-pathological tail chasing, and to identify the most reliable indicators of whether the behavior is of welfare concern. We hope that this and other similar projects will provide the basis of an epidemiologic study for deeper understanding which will establish the basis for prevention and control of these kinds of behavioral problems.
